# Development of a Novel Reference Plasmid for Accurate Quantification of Genetically Modified Kefeng6 Rice DNA in Food and Feed Samples

**DOI:** 10.1155/2013/134675

**Published:** 2013-11-13

**Authors:** Liang Li, Xiujie Zhang, Yusong Wan, Wujun Jin

**Affiliations:** Biotechnology Research Institute, Chinese Agricultural Academy of Sciences, No. 12 Zhongguancun South Street, Haidian District, Beijing 100081, China

## Abstract

Reference plasmids are an essential tool for the quantification of genetically modified (GM) events. Quantitative real-time PCR (qPCR) is the most commonly used method to characterize and quantify reference plasmids. However, the precision of this method is often limited by calibration curves, and qPCR data can be affected by matrix differences between the standards and samples. Here, we describe a digital PCR (dPCR) approach that can be used to accurately measure the novel reference plasmid pKefeng6 and quantify the unauthorized variety of GM rice Kefeng6, eliminating the issues associated with matrix effects in calibration curves. The pKefeng6 plasmid was used as a calibrant for the quantification of Kefeng6 rice by determining the copy numbers of event- (77 bp) and taxon-specific (68 bp) fragments, their ratios, and their concentrations. The plasmid was diluted to five different concentrations. The third sample (S3) was optimized for the quantification range of dPCR according to previous reports. The ratio between the two fragments was 1.005, which closely approximated the value certified by sequencing, and the concentration was found to be 792 copies/**μ**L. This method was precise, with an RSD of ~3%. These findings demonstrate the advantages of using the dPCR method to characterize reference materials.

## 1. Introduction

Genetically modified organisms (GMOs) are widely used and have been planted in over 28 countries. The hectares occupied by GMOs have increased by an unprecedented 100-fold, from 1.7 million hectares in 1996 to 170 million hectares in 2012 [1]. A total of 319 events for 25 crops have been approved for commercialization, with maize accounting for most approved events (121), followed by cotton (48), potato (31), canola (30), and soybean (22). Due to the rapid increase in the number of biotech crops used for food production, consumers are becoming increasingly concerned about the health risks posed by GM crops and their derivatives. These concerns have resulted in GMO-labeling regulations in over 40 countries, and the sale of food and feed that contain unauthorized GMOs is prohibited in certain markets [2].

Ensuring that food and feed are correctly labeled to indicate whether ingredients are derived from GMOs is a considerable issue facing manufacturers, retailers, and enforcement agencies [3]. In October 2004, the European Commission recommended that the GM content of food and feed can be expressed as the percentage of GM DNA copy numbers in relation to target taxon-specific DNA copy numbers calculated in terms of haploid genomes [4]. As a consequence, reference materials are needed for the evaluation of copy number ratio between transgenic and taxon-specific genes.

Among DNA-based approaches for determining food authenticity, quantitative real-time PCR (qPCR) is often considered the gold standard for nucleic acid quantification [5–7], and it is the most commonly used technique for analyzing the presence of nucleic acids in food and feed samples. However, qPCR is not well suited for the measurement of absolute concentrations because its precision is limited (~20%) and it often performs poorly in regard to low-copy number templates [8]. In addition, the use of a calibration curve and reference materials often means that the evaluation of the GM content of the unknown sample is subject to “matrix effects” [9].

The emerging digital platform technique offers a unique advantage over conventional qPCR for measuring nucleic acids that may be particularly susceptible to the previously challenges. Digital PCR (dPCR) amplifies a single DNA template from minimally diluted samples, thereby generating amplicons that are derived exclusively from one positive reaction chamber that contains at least one target molecule. In highly diluted samples, the number of positive wells is equal to the number of target molecules. Using a Poisson-based algorithm, template abundance can be calculated with a 95% confidence interval, even if a large proportion of the reactions are positive and contain more than one target molecule [10]. Thus, dPCR transforms the exponential, analog, and single-molecule sensitivity of classic PCR into a linear, digital signal [11–13]. dPCR performed using microfluidics could have a major impact on clinical diagnosis [14–18], single-cell expression analyses [19–21], next-generation sequencing [22–25], and GMO analysis [9, 26], among other processes.

The presence of unauthorized GMOs in food and feed samples may increase in the near future. To enable enforcement laboratories to continue detecting all GM events and identify unauthorized GMOs in food and feed samples, intensive screening is required [27]. It is necessary for the European Union (EU) to reinforce controls on the import of Chinese rice products entering the EU market, because certain Chinese rice imports have been contaminated with unauthorized GMOs, including the rice strains BT63, Kefeng6, and Kemingdao 1. 

This paper reports the construction of a novel plasmid for use as a calibrant to quantify Kefeng6 rice, which contains both the *Bt* and *CpTI* transgenes (*Bt/CpTI*). We applied a novel dPCR platform, the BioMark system (Fluidigm, South San Francisco, CA, USA), which allows for the simultaneous amplification of thousands of PCR samples and requires as little as 1 ng of DNA per sample. PCR products generated on the BioMark Access Array system were used to quantify the ratio and copy number concentration of the pKefeng6 plasmid.

## 2. Materials and Methods

### 2.1. Plant Materials and Genomic DNA Isolation

Rice Kefeng6 flour samples were supplied by the China National Rice Research Institute. Genomic DNA from Kefeng6 was isolated from rice flour using the Wizard Magnetic DNA Purification System for Food according to the manufacturer's instructions (Promega, Madison, WI, USA). The DNA pellets were dissolved in 100 *μ*L nuclease-free water. DNA quantification was performed using a PicoGreen assay (Quant-iT PicoGreen dsDNA Kit; Invitrogen Carlsbad, CA, USA), and the samples were diluted to a 50 ng/*μ*L working stock, which was stored in aliquots at −80°C.

### 2.2. Construction and Purification of the pKefeng6 Plasmid DNA

The pKefeng6 plasmid, which was constructed based on pEASY-T3 (TransGen, Beijing, China), was cloned using overlapping PCR of a sequence that included a 68 bp fragment of *gos9* and a 77 bp GM event-specific fragment of Kefeng6. The primers used for plasmid construction are listed in Table 1. The two fragments were amplified using genomic DNA from Kefeng6 as the template and the following primers KF6-1F, KF6-1R, gos9-1F, and gos9-1R. Complementary primers (Fusion-F and Fusion-R) and PCR were used to generate two DNA fragments with overlapping ends. These fragments were combined to generate a fusion product. Digestion of the *Bam*HI and *Hin*dIII sites was performed to verify the identities of the fragments. To confirm the sequences of the inserted fragments, SinoGenoMax Co., Ltd., Sangon Co., Ltd., and Invitrogen Co., Ltd. performed direct sequencing.

JM109-competent cells containing the correct inserts were cultured and the plasmid DNA was isolated and purified using the PureYield Plasmid Midiprep System (Promega) according to the manufacturer's protocol. Plasmid DNA concentrations (*μ*g/mL) were measured using the PicoGreen assay and calculated as copy numbers of pKefeng6, considering the size of the plasmid and the molecular weight of the dsDNA. The pellet was air dried and dissolved in 30 *μ*L of 1 × TE_0.1_ (10 mM Tris, 0.1 mM EDTA, and pH 8.0). 

A plasmid solution containing 3.71 × 10^5^ cp/*μ*L was prepared by diluting the stock plasmid solution in a buffer containing 1 mM Tris, 0.01 mM EDTA, and pEasy-T3 plasmid DNA at pH 8.0. The plasmid was serially diluted at 1 : 10, 1 : 10, 1 : 5, 1 : 5, and 1 : 5 to obtain qPCR samples with the expected number of plasmid copies. The five diluted plasmid DNA samples (S1: 37100, S2: 3710, S3: 742, S4: 148, and S5: 30 copies per reaction in 5 *μ*L) were used to construct a standard curve. One of the five diluted samples (S3) was then used for absolute quantification by dPCR.

### 2.3. Oligonucleotide Primers and Probes

Invitrogen, Inc. (Shanghai, China) synthesized the oligonucleotide primers and TaqMan probes used for quantification (Table 1). To amplify the transgene border junctions of Kefeng6, we used a previously described protocol [28]. The endogenous fragments of* gos9* were amplified using a quantitative PCR amplification protocol [29].

### 2.4. Quantitative Real-Time PCR

DNA amplification and data collection were performed using the LightCycler 480 II. All reactions were performed with the TaqMan Universal Master Mix (Applied Biosystems, CA, USA) following the manufacturer's instructions using the 25 *μ*L reaction solution described in Table 2. A no-template control (NTC) consisting of deionized H_2_O was prepared as a negative control. To minimize pipetting error, all components except the DNA were premixed, and the final reaction mix was prepared gravimetrically by combining the DNA solution and the premixed solution [30]. To generate standard curves, the plasmids were serially (1 : 10, 1 : 10, 1 : 5, 1 : 5, and 1 : 5) diluted in Axgen tubes over the appropriate concentration range. To achieve a reliable curve for each measured parameter, the plasmids were PCR amplified in 3 replicates for each standard dilution point over the complete standard curve range. The reaction conditions used the following PCR step-cycle program: 50°C for 2 min, 95°C for 10 min, 45 cycles of 95°C for 30 s, and 60°C for 1 min. The raw data were analyzed using a LightCycler SW 1.5.

### 2.5. Digital PCR Procedure

The Fluidigm digital array is a novel nanofluidic biochip for dPCR reactions [20]. Utilizing nanoscale valves and pumps, this digital array delivers up to 12 mixtures of samples and PCR reagents into 12 individual panels. Each panel contains 765 independent 6 nL chambers. This nanofluidic platform utilizes soft lithography and silicone rubber to create nanoscale valves and pumps that can be used in serial or parallel applications [10]. 

We performed all digital experiments on the BioMark System using the 12.765 Digital Array (Fluidigm, South San Francisco, CA, USA). Reaction mixtures (10 *μ*L) were prepared for each panel, as shown in Table 2, and approximately 4.6 *μ*L of this reaction mixture was distributed throughout the partitions within each panel using the Fluidigm IPC Controller (Fluidigm, South San Francisco, CA, USA). The thermocycling conditions included a 10 min hot start at 95°C, followed by 45 cycles of two-step PCR, which consisted of 15 s at 95°C for denaturation and 1 min at 60°C for annealing and extension. Molecules of the two fragments were amplified independently. After the reaction was completed, the raw data were processed using the Fluidigm Digital PCR Analysis software with a manually set threshold of 0.65 and a target Cq range of 20 to 35.

## 3. Results and Analysis

### 3.1. Construction of pKefeng6

**Figure 1:**
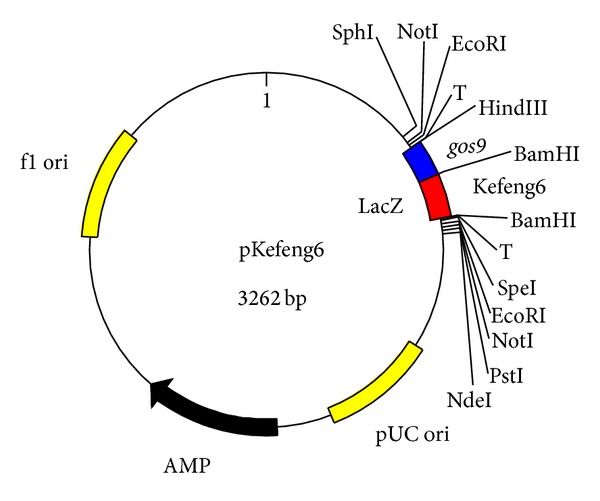
Schematic diagram of the fragments integrated into pKefeng6. *gos9*: fragment of the endogenous rice reference gene *gos9*; Kefeng6: event-specific fragment from Kefeng6 rice.

In this study, we constructed a pKefeng6 plasmid that contained two targets, a BT event and *gos9*. The pKefeng6 plasmid was designed as a positive calibrator for Kefeng6 rice. In pKefeng6 (Figure 1), the exogenous rice fragments and endogenous gene fragments were spliced by inserting a *Bam*HI site, which was connected by overlapping PCR and cloned into the vector. Sequencing results from three different labs (data not shown) verified that the expected plasmids were obtained.

### 3.2. Calibration Curves

**Figure 2:**
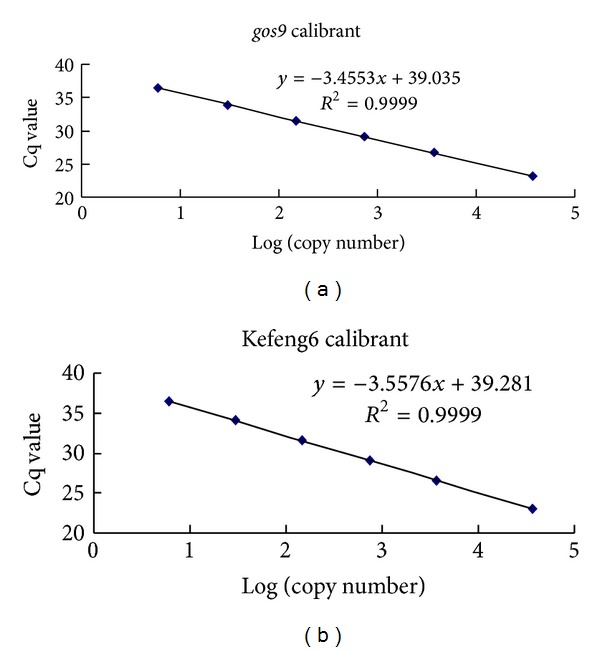
Calibration curves. Sample calibration curves associated with the estimations of the endogenous copy number ((a), *gos9*) and the transgenic copy number ((b), Kefeng6). The *x*-axis represents the logarithm of the estimated copy number of the calibrant, and the *y*-axis represents the Cq value.

Purified plasmid DNA was used to prepare the standard curves. The linear regression equations and associated Pearson's correlation coefficients (*R* ^2^) for the three qPCR runs are illustrated in Figure 2. The regression correlation coefficients of the standard curves were 0.9999 for both *gos9* and Kefeng6. PCR reaction efficiencies were generated based on the equation *E* = 10^−1/slope^ − 1 and the values were 91.3% and 94.9% for the *gos9* and Kefeng6-specific fragments, respectively, indicating highly efficient reactions. Importantly, the assays optimized for qPCR efficiency played an important role in the absolute quantification of dPCR.

### 3.3. Quantification of pKefeng6

**Figure 3:**
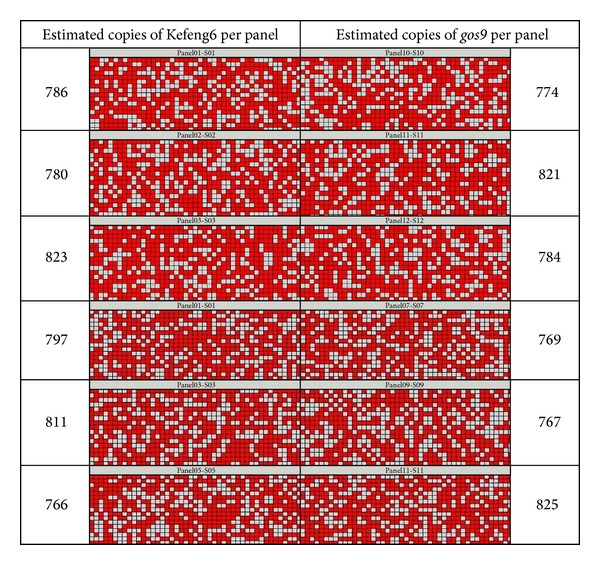
Heat map of S3 dilution. Red spots indicate reactions in which amplification occurred, and gray spots represent reactions with no observable amplification. Estimated copy numbers of the two fragments, which were calculated using digital PCR (including of Poisson transformations), are shown on the left or right of each heat map.

The precision of the copy number determination of pKefeng6 was investigated using the 12.765 dPCR chip. The dPCR reactions were repeated six times, with three parallel panels per chip. Typical heat maps for Kefeng6 and *gos9* are presented in Figure 3. The partitions were marked as positive (shown in red) if the target DNA molecule was amplified and a fluorescent signal above a manually set threshold was detected. The estimated copy numbers are summarized in Table 3. In the same sample, the estimated Kefeng6 copy numbers were higher than the *gos9* copy numbers. However, the Kefeng6 and *gos9* copy numbers were not significantly different according to two-sided *t*-tests that assumed unequal variances (*P* = 0.786 > 0.05). The results for the Kefeng6 and the *gos9 *(*n* = 6) demonstrated the absence of bias resulting from a specific set of primers and probes. The ratio of Kefeng6-specific DNA to *gos9* fragments in pKefeng6 was 1.005, which was considered a good approximation (data not shown). The mean values of both PCR targets (*n* = 6) were considered to be one independent measurement. The mean copy number concentration of S3 was 792 with a relative standard deviation (RSD) of 2.87%, which may be contributed by random distribution of the target molecules throughout the partitions and consistent amplification from single molecules [30].

To obtain the most accurate possible measurement using this platform, dPCR should be performed at 200–700 positive partitions per 765 chamber panel [31]. In this study, the dilution S3 was expected to lie within the manufacturer-recommended quantitation range of 446–516 positive partitions per panel. The other four dilutions could be calculated by the concentration of S3 according to the chamber range. PicoGreen measurements were prepared alongside the dPCR studies for comparison. The estimated copy numbers were higher than the expected numbers, as previously described [32]. A recent publication described the tendency for digital arrays to overestimate the copy numbers of transgenes compared to the expected numbers when the expected copy number was 158 or greater. This tendency was reversed for expected copy numbers of 79 or lower [9]. The expected copy number in dilution S3 was 742, and the estimated copy numbers were 794 and 790, consistent with the reported pattern.

## 4. Discussion

The number of GMOs cultivated worldwide for commercial or research purposes continues to increase. This continued increase will add to the complexity of the efforts of enforcement laboratories to detect not only authorized GMOs in food and feed samples but also unauthorized GMOs, which are also expected to steadily increase in the coming years [27]. Genomic or plasmid DNA can be used as analyte standards. Genomic DNA must first be extracted from a matrix, making it susceptible to matrix effects and processing influences such as degradation [33]. The advantages of using plasmid standards include their ease of preparation, low cost, universal applicability, and long-term stability. These advantages make plasmid DNA more attractive than genomic DNA standards from certified reference materials (CRMs) [34]. To enforce GMO-labeling regulations, GMOs must be quantitatively identified, requiring the use of reference molecules as calibrants [35]. In this study, we developed the novel plasmid pKefeng6, which can be used as a calibrant for the identification and quantification of unauthorized Kefeng6 rice or its derivatives. 

The gold standard method of estimating DNA concentration via qPCR is quantification cycle (Cq) standard curve quantification, which requires the time- and labor-intensive construction of a standard curve. However, dPCR has the potential to provide absolute quantification instead of relative quantification. In addition, this method can overcome the lack of suitable standards that are available for constructing a calibration curve and reduce the associated “matrix effects” that are often associated with qPCR approaches. 

Following the method acceptance criteria set by the European Network of GMO Laboratories (ENGL), the accuracy should be within ±25% of the accepted reference value over the whole dynamic range. The accuracy of the dPCR system (1.005) is highly closeness of measurements of a quantity to that quantity's true value (1.000) by sequencing. As this technique uses quantification of each of the targets of interest, it could be more accurate at detecting the subtle bias. The dPCR technique has been demonstrated to be both accurate and highly reproducible compared to qPCR. The RSD of the copy number ratio and concentration for the dPCR assay was determined to be ~3.0%, which was significantly lower than the previously reported RSD value of approximately 20% [8]. Because this digital assay does not rely on internal or external standards, the RSD of approximately 3.0% demonstrates the true accuracy of the assay. Importantly, dPCR measurements are performed without using any calibration agent; thus, this technique may be considered a primary method for the certification of nucleic acid reference materials. The measurement principle behind dPCR has a high metrological quality [26].

## 5. Conclusions

The novel plasmid constructed in this study is suitable for the detection and quantification of Kefeng6 GM rice and feed products containing this DNA fragment. Additionally, the quantification of pKefeng6 by dPCR can be applied to qPCR for the management of GMO product labeling with a high level of accuracy and precision. dPCR is more expensive per sample than classical qPCR; therefore, the practicality of using dPCR as a routine method for GMO analysis requires further investigation. However, this novel technology can be used to validate event-specific methods and certify the values of calibrants. This work provides the essential technical basis for developing this method into an official standard method.

## Figures and Tables

**Table 1:** Primers and probes used in this study.

Target	Purpose	Primer sequence (5′→3′)	Amplicon size
Kefeng6	Construction	KF6-1F **GGATCC**ACGTAGTACGTACCGCCGTG^1^	89
		KF6-1R **GGATCC**AGTGCAGATGCATGAATCGC^1^	
	Quantification	KF6-2F ACGTAGTACGTACCGCCGTG	77
		KF6-2R AGTGCAGATGCATGAATCGC	
		KF6-P FAM-CCGCGCGTTGTACTGAGAACCA-TAMRA	

*gos9 *	Construction	*gos9*-1F **GGATCC**TTAGCCTCCCGCTGCAGA^1^	80
		* gos9*-1R **AAGCTT**AGAGTCCACAAGTGCTCCCG^2^	
	Quantification	*gos9*-2F TTAGCCTCCCGCTGCAGA	68
		*gos9*-2R AGAGTCCACAAGTGCTCCCG	
		*gos9*-P FAM-CGGCAGTGTGGTTGGTTTCTTCGG-TAMRA	

Kefeng6 and *gos9 *	Fusion	Fusion-F GGGAGGCTAAGGATCCAGTGCAGATG	183
		Fusion-R CATCTGCACTGGATCCTTAGCCTCCC	

^1^ *Bam*HI site in bold type, ^2^ *Hin*dIII site in bold type.

**Table 2:** Optimized concentrations for qPCR and dPCR assays.

Component	qPCR		dPCR	
	Kefeng6 (*μ*L)	*gos9* (*μ*L)	Kefeng6 (*μ*L)	*gos9* (*μ*L)
TaqMan Universal Master Mix (2×)	12.5	12.5	5	5
Primer-F (10 *µ*M)	1	1	0.4	0.4
Primer-R (10 *µ*M)	1	1	0.4	0.4
Probe-P (10 *µ*M)	0.5	0.5	0.2	0.2
Template (~20 ng/*μ*L)	5	5	2	2
Loading (10×)	0	0	1	1
H_2_O	5	5	1	1

Total	25	25	10	10

**Table 3:** Copy number concentrations and ratios of the third sample (S3) as determined by dPCR.

Kefeng6				*gos9*				Concentration		Ratio	*P*
Positive partitions	Estimated copies	Average Number	RSD (%)	Positive partitions	Estimated copies	Average number	RSD (%)	Mean (cp/*µ*L)	RSD (%)	KF6/*gos9*	
494	786	794	2.6	476	774	790	3.3	792	2.87	1.005	0.786
471	780			494	821						
516	823			492	784						
465	797			455	769						
481	811			446	767						
455	766			488	825						

The average number was calculated from six replicate measurements with three parallel panels per chip (*n* = 6), and this value was adjusted to account for the gravimetrically prepared PCR solutions used in the Kefeng6 and *gos9* assays. RSD: relative standard deviation; *P*: probability.

## References

[1] James C (2013). *Global Status of Commercialized Biotech/GM Crops: 2012*.

[2] Yang L, Guo J, Pan A (2007). Event-specific quantitative detection of nine genetically modified maizes using one novel standard reference molecule. *Journal of Agricultural and Food Chemistry*.

[3] Burrell A, Foy C, Burns M (2011). Applicability of three alternative instruments for food authenticity analysis: GMO identification. *Biotechnology Research International*.

[4] European Commission (2004). Recommendation 2004/787/EC of 4 October 2004 on technical guidance for sampling and detection of genetically modified organisms and material produced from genetically modified organisms as or in products in the context of Regulation (EC) No. 1830/2003. *Official Journal of the European Union*.

[5] Heid CA, Stevens J, Livak KJ, Williams PM (1996). Real time quantitative PCR. *Genome Research*.

[6] Livak KJ, Schmittgen TD (2001). Analysis of relative gene expression data using real-time quantitative PCR and the 2-ΔΔCT method. *Methods*.

[7] Schmittgen TD, Lee EJ, Jiang J (2008). Real-time PCR quantification of precursor and mature microRNA. *Methods*.

[8] Heyries KA, Tropini C, Vaninsberghe M (2011). Megapixel digital PCR. *Nature Methods*.

[9] Burns MJ, Burrell AM, Foy CA (2010). The applicability of digital PCR for the assessment of detection limits in GMO analysis. *European Food Research and Technology*.

[10] Dube S, Qin J, Ramakrishnan R (2008). Mathematical analysis of copy number variation in a DNA sample using digital PCR on a nanofluidic device. *PLoS ONE*.

[11] Vogelstein B, Kinzler KW (1999). Digital PCR. *Proceedings of the National Academy of Sciences of the United States of America*.

[12] Pohl G, Shih L-M (2004). Principle and applications of digital PCR. *Expert Review of Molecular Diagnostics*.

[13] Sanders R, Huggett JF, Bushell CA, Cowen S, Scott DJ, Foy CA (2011). Evaluation of digital PCR for absolute DNA quantification. *Analytical Chemistry*.

[14] Diehl F, Li M, Dressman D (2005). Detection and quantification of mutations in the plasma of patients with colorectal tumors. *Proceedings of the National Academy of Sciences of the United States of America*.

[15] Oehler VG, Qin J, Ramakrishnan R (2009). Absolute quantitative detection of ABL tyrosine kinase domain point mutations in chronic myeloid leukemia using a novel nanofluidic platform and mutation-specific PCR. *Leukemia*.

[16] Lo YMD, Lun FMF, Chan KCA (2007). Digital PCR for the molecular detection of fetal chromosomal aneuploidy. *Proceedings of the National Academy of Sciences of the United States of America*.

[17] Lo YMD, Tsui NBY, Chiu RWK (2007). Plasma placental RNA allelic ratio permits noninvasive prenatal chromosomal aneuploidy detection. *Nature Medicine*.

[18] Lo YMD, Chiu RWK (2008). Noninvasive prenatal diagnosis of fetal chromosomal aneuploidies by maternal plasma nucleic acid analysis. *Clinical Chemistry*.

[19] Guo G, Huss M, Tong GQ (2010). Resolution of cell fate decisions revealed by single-cell gene expression analysis from zygote to blastocyst. *Developmental Cell*.

[20] Spurgeon SL, Jones RC, Ramakrishnan R (2008). High throughput gene expression measurement with real time PCR in a microfluidic dynamic array. *PLoS ONE*.

[21] Warren L, Bryder D, Weissman IL, Quake SR (2006). Transcription factor profiling in individual hematopoietic progenitors by digital RT-PCR. *Proceedings of the National Academy of Sciences of the United States of America*.

[22] Jones MA, Bhide S, Chin E (2011). Targeted polymerase chain reaction-based enrichment and next generation sequencing for diagnostic testing of congenital disorders of glycosylation. *Genetics in Medicine*.

[23] Kim H, Bartsch MS, Renzi RF (2011). Automated digital microfluidic sample preparation for next-generation DNA sequencing. *Journal of Laboratory Automation*.

[24] White RA, Blainey PC, Fan HC, Quake SR (2009). Digital PCR provides sensitive and absolute calibration for high throughput sequencing. *BMC Genomics*.

[25] Zernant J, Schubert C, Im KM (2011). Analysis of the ABCA4 gene by next-generation sequencing. *Investigative Ophthalmology & Visual Science*.

[26] Corbisier P, Bhat S, Partis L, Rui Dan Xie V, Emslie KR (2010). Absolute quantification of genetically modified MON810 maize (*Zea mays* L.) by digital polymerase chain reaction. *Analytical and Bioanalytical Chemistry*.

[27] Broeders SRM, De Keersmaecker SCJ, Roosens NHC (2012). How to deal with the upcoming challenges in GMO detection in food and feed. *Journal of Biomedicine and Biotechnology*.

[28] Huang X, Zhang Y, Hou L, Zhang Q, Chen H, Zhu S (2010). The quantitative real-time PCR detection of genetically modified rice kefeng No. 6. *Biotechnology Bulletin*.

[29] Hernández M, Esteve T, Pla M (2005). Real-time polymerase chain reaction based assays for quantitative detection of barley, rice, sunflower, and wheat. *Journal of Agricultural and Food Chemistry*.

[30] Bhat S, Herrmann J, Armishaw P, Corbisier P, Emslie KR (2009). Single molecule detection in nanofluidic digital array enables accurate measurement of DNA copy number. *Analytical and Bioanalytical Chemistry*.

[31] BioMark BioMark advanced development protocol 10. absolute quantitation using the digital array.

[32] Bhat S, Curach N, Mostyn T, Bains GS, Griffiths KR, Emslie KR (2010). Comparison of methods for accurate quantification of DNA mass concentration with traceability to the international system of units. *Analytical Chemistry*.

[33] Chaouachi M, Alaya A, Ali IBH (2013). Development of real-time PCR method for the detection and the quantification of a new endogenous reference gene in sugar beet “*Beta vulgaris* L.”: GMO application. *Plant Cell Reports*.

[34] Wang X, Teng D, Yang Y, Tian F, Guan Q, Wang J (2011). Construction of a reference plasmid molecule containing eight targets for the detection of genetically modified crops. *Applied Microbiology and Biotechnology*.

[35] Huang C-C, Pan T-M (2005). Event-specific real-time detection and quantification of genetically modified roundup ready soybean. *Journal of Agricultural and Food Chemistry*.

